# Anti-cancer effects of frankincense methanolic extract on brain metastatic breast cancer cells

**DOI:** 10.22038/AJP.2024.24655

**Published:** 2025

**Authors:** Mohammad Kamalabadi-Farahani, Fatemeh Jamshidi Adegani, Roqaye Karimi, Amir Atashi

**Affiliations:** 1 *Department of Tissue Engineering, School of Medicine, Shahroud University of Medical Sciences,* *Shahroud, Iran*; 2 *Laboratory for Stem Cell & Regenerative Medicine, Natural and Medical Sciences Research Center, University of Nizwa, Nizwa, P. O. Box: 33, PC 616, Oman*; 3 *Department of Hematology and Cell Therapy, Faculty of Medical Sciences, Tarbiat Modares University, Tehran, Iran*; 4 *Department of Medical Laboratory Sciences, School of Allied Medical Sciences, Shahroud University of Medical Sciences, Shahroud, Iran*

**Keywords:** Frankincense, Brain metastasis, Triple negative breast cancer, Antimetastatic

## Abstract

**Objective:**

Brain metastasis in patients with breast cancer is considered a deadly event. The oleogum resins of Boswellia species, known as Frankincense has been found to have anti-cancer properties in many studies. The main purpose of our research was to evaluate these effects on brain metastatic cancer cells.

**Materials and Methods:**

Primary (4T1T) and brain metastatic (4T1B) tumor cells were isolated from breast cancerous mice. Cytotoxic, apoptotic, and anti-metastatic effects of the methanolic extract of frankincense were evaluated with MTT assay, propidium iodide (PI) flow cytometric assay, and scratch test, respectively. Zymography assay was used to evaluate the effects of extract on the matrix metalloproteinases-2 and -9 (MMP-2 /9) expression/activity.

**Results:**

The methanolic extract of frankincense has significant cytotoxic and apoptotic effects on 4T1B cells (p<0.001). Interestingly, 4T1B cells are more prone to these effects than 4T1T cells. In 4T1B, the anti-metastatic effects of frankincense extract were confirmed. Frankincense suppressed MMP-2/9 protein expression both in 4T1T and 4T1B.

**Conclusion:**

Our findings indicated that frankincense extract has a potent cytotoxic effect on brain metastatic tumor cells and induces apoptosis in these cells. Unlike many anti-cancer drugs which have very little ability to combat and kill brain metastatic cancer cells, frankincense extract can be considered a suitable candidate to fight these cells.

## Introduction

Breast cancer is a very common cancer among women globally (25.1% of all cancers)(Siegel et al., 2023). Approximately 12% of diagnosed cases finally turn into metastatic form (Rashid et al., 2023). Among all subtypes, triple-negative breast cancer (TNBC) lacks three important breast cancer receptors (estrogen receptor, progesterone receptor, and HER2) which play an essential role in tumorigenesis and resistance to treatment (Wang et al., 2019). The prevalence of brain metastasis (BM) is high in breast cancer patients, especially in those with TNBC. Also, 10 – 16% of women with breast cancer eventually face BM. Patients with breast cancer BM have the median survival of 8.3 months (Sun, Xu et al., 2023).

The main challenge in the treatment of TNBC-BM is the lack of targeted treatment options and problems related to drug delivery to the brain. Current medication in metastatic cases is chemotherapy which may cause serious side effects (Isakoff, 2010). Research using patient samples and modeling of BM in animal models has led to a better understanding of the pathobiology of BM (Bos et al., 2009; Hall and Stoica, 1994). 

Many efforts have been made on natural therapeutic components in order to discover new medications and pave the way to introduce a remedy for incurable disease. It has been reported that the oleogum resins of *Boswellia* species known as frankincense has anti-cancer properties produced via several mechanisms including immune cell regulation and leukotriene synthesis inhibition (Efferth and Oesch, 2022). Boswellic acids, major constituents of frankincense, are able to inhibit proinflammatory cytokines and have toxicity against TNBC cell lines. Current findings reveal that boswellic acids induce cell proliferation arrest and inhibition of angiogenesis, invasion and metastasis in breast cancer murine models (Al-yasiry and Kiczorowska, 2016). 

TNBC treatment is still a challenging issue. On the other hand, our failure in TNBC treatment leads to disease progression and metastasis. Therefore, there is an immediate need to develop effective and novel treatment strategies. Several authors have emphasized on anti-cancer properties of frankincense, but these effects on brain metastatic tumor cells have not been investigated previously. Here, we report the anti-cancer effects of frankincense extract on BM cancer cells.

## Materials and Methods

### Syngeneic modeling of metastatic TNBC in mice

For tumor induction in female BALB/c mice, 10^5^ 4T1 cells suspended in 100 microliters (μl) phosphate buffered saline (PBS), were subcutaneously injected into the mice flanks. For extraction of primary and metastatic tumor cells after 35 days of tumor induction in mice, the primary tumor and brain were surgically removed, and surface blood was washed away using PBS. After mechanical digestion, the main tumor and the brain were digested using enzymatic methods in 10 mg/ml collagenase type IV at 37°C for 75 min on a platform rocker. The enzymes were all bought from Sigma (St Louis, MO, USA). The digested organs were rinsed with PBS and passed through 70-um cell strainers. The following stage involved resuspending cleaned cells in media containing 10% fetal bovine serum (FBS), 100 units per milliliter of penicillin, and 100 ug/ml of streptomycin (all from Gibco, USA). In the end, the cells were grown in 5% CO_2_ at 37°C. The Ethics Committee of Shahroud University of Medical Sciences approved this study for ethics in animal research (registration number: IR.SHMU.REC.1400.028). 

### Frankincense extract preparation

In recent years, several publications have emphasized that polar extracts of plants have strong anti-cancer effects (Alipanah and Zareian, 2018), accordingly, we used methanol extract in our work. The dried powder of *Boswellia sacra* (5 g) (collected from Oman) was extracted in 50 ml of methanol for 48 hr at 25°C temperature on a shaker and the extract was then filtered with Whatman grade 1 qualitative filter paper. In the next step, the filtrates collected from methanolic extraction were subjected in an oven at 45°C to remove the entire methanol. Samples was dissolved in dimethyl sulfoxide (DMSO) (Merck, Germany) under sterile conditions and kept at -20°C until use.

### MTT colorimetric assay

In this part, 1×10^4^ brain metastatic tumor cells were seeded in 96-well polystyrene culture plates. Following overnight incubation, cells were treated in triplicate with 20, 40, 80, 100, 150 and 200 μg/ml concentrations of the extract and the plate was incubated at 37°C. After a 48-h incubation, medium was replaced by 100 ml of MTT solution (concentration 5 mg/ml, Sigma). After 4 hr, the supernatant medium was removed and 50 ml of DMSO was added to each well to dissolve the colored precipitate of formazan. In the end, the optical absorbance of each well was measured using an ELISA Plate Reader at 570 nm. For comparison MTT assay was done on primary tumor cell in the same way.

### Apoptosis assay

Primary and metastatic breast cancer cells, similar to the prior step, were prepared and treated with IC_50_ concentration of extract for 48 hr in triplicate. To measure cell apoptosis, MabTag's annexin-V apoptosis detection kit (Biolegend, Cat No. 640914) was used to perform cell apoptosis assays according to the manufacturer's protocol. Flow cytometric analysis was performed with FlowJo software, version 7.2.2.

### Wound scratch assay

In this assay, 12-well plates were used to seed primary and metastatic breast cancer cells. After cell seeding, the cells were incubated at 37°C until confluent and monolayer formation. After incubation, a physical scratch was made on a confluent cell monolayer, and free cells were eliminated from the scratched region by washing. Afterwards, cells were preserved with or without the extract in serum-containing medium overnight at 37°C, and migration of cells between scratch areas was imaged at 0 and 24 hr using an inverted microscope (Olympus CK40, Tokyo, Japan) in each well (Sun et al., 2016).

### Gelatin zymography

In this step, 9% polyacrylamide gel was simultaneously polymerized with gelatin (0.5 mg/ml) as substrate and 100 μl of the samples were loaded in each well of the gel. Following electrophoresis, gels were washed twice in 1% Triton X-100 for 30 min to remove sodium dodecyl sulfate (SDS) and then, incubated in developing buffer (50 mM TrisHCl, 0.15 M NaCl, 5 mM CaCl_2_) with pH 7.8 at 37°C for 48 hr. In the end, gels were stained with Coomassie blue. Decolorization of gels was done in several steps and the areas resulting from the interaction of gelatinase with gelatin appeared as colorless areas on a black background.

### Statistical analysis

All statistical analyses were performed by t-test in Graphpad Prism, and a p-value ≤0.01 was considered significant. All assays were carried out in triplicate and results are shown as mean±SD.

## Results

### Primary and brain metastatic tumor cell culture

**Figure 1:**
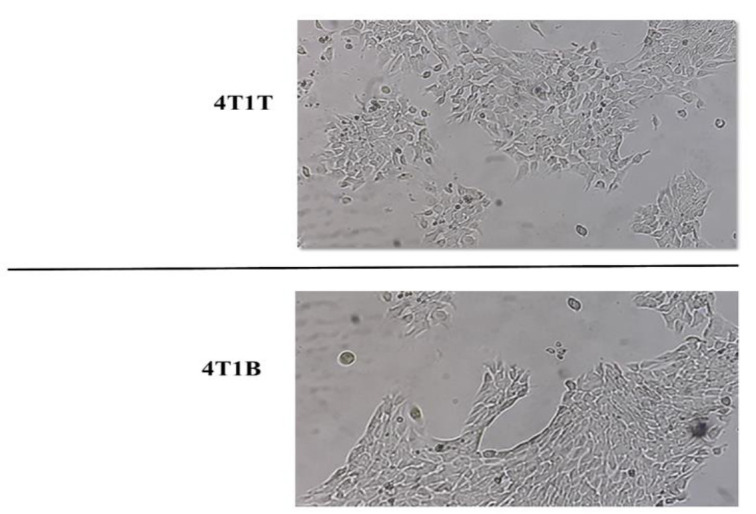
Primary and brain metastatic tumor cells culture. Primary (4T1T) and brain metastatic (4T1B) tumor cell cultivation and propagation in cell culture condition. After 3 passages, the tumor cells were ready for molecular and cellular analysis.

The primary tumor cells, called 4T1T and the brain metastatic tumor cells, called 4T1B cells, formed colonies in the culture medium after initial isolation and were purified after 3 consecutive passages (Figure 1).

### Cytotoxic effects of frankincense extract against metastatic tumor cells

**Figure 2:**
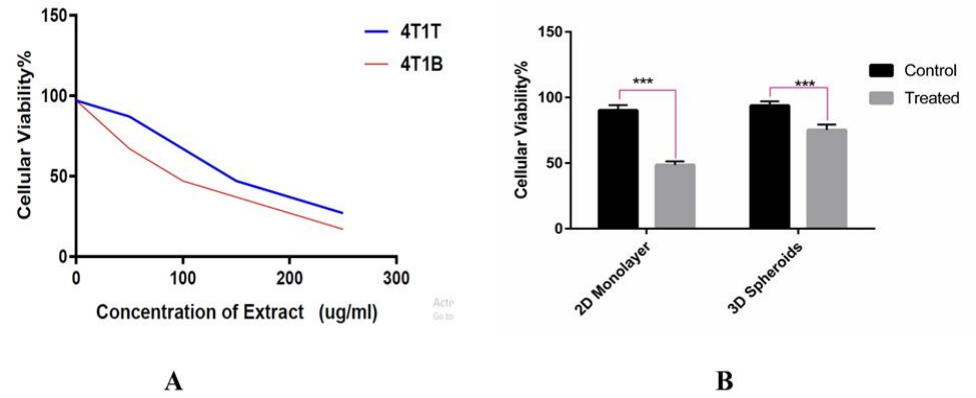
Cytotoxic assessment of frankincense extract on primary and brain metastatic tumor cells by MTT assay. (A) Exposing of 4T1T and 4T1B to extract resulted in a significant decrease in cells viability in a dose-dependent manner. For 4T1T and 4T1B, the IC_50_ value was found to be 105 and 85 μg/ml respectively. (B)The results indicated that compared with 4T1T, 4T1B are more susceptible to cytotoxic effects of the extract (T test is *used* for statistical analysis and ***p<0.001).

Cytotoxic effects of frankincense extract on 4T1T and 4T1B cells were measured using MTT method.. Initial evaluations showed that these effects were evident after 48 hours of exposure to the extract. Therefore, exposing the cells to frankincense extract led to a considerable reduction in cells viability in a dose-dependent manner (p<0.001, Figure 2). For 4T1T, the value of IC_50_ of the extract using MTT assay was 105 μg/ml, which caused the death of 50% of cancer cells compared to the control group. In 4T1B cells, as shown in Figure 2, exposing the cells to frankincense extract led to a considerable reduction in cells viability. Surprisingly, the results proved that compared with 4T1T, 4T1B are more susceptible to cytotoxic effects of the extract. For 4T1B, the value of IC_50_ of the extract using MTT assay was 85 μg/ml.

### Apoptotic effects of frankincense extract on 4T1B

**Figure 3:**
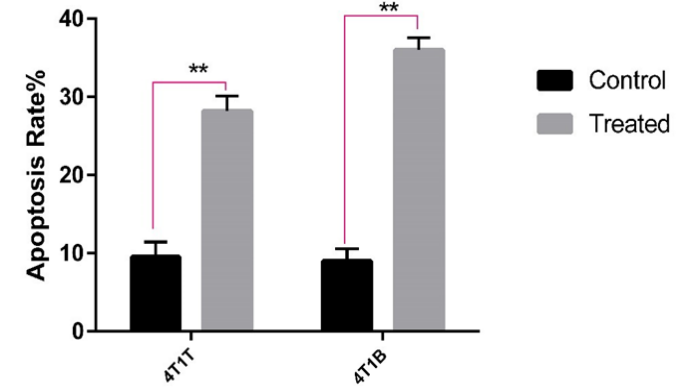
Frankincense regulated apoptosis in 4T1T and 4T1B. 4T1T and 4T1B were treated with frankincense extract (105 μg/ml) for 48 hr. Cell apoptosis was analyzed by flow cytometry. The level of apoptosis was 28% in 4T1T but 36% in 4T1B (T test is *used* for statistical analysis and **p<0.01).

To determine the apoptotic effects of the extract, annexin V kit was used. For this purpose, both 4T1T and 4T1B cells were treated with a concentration of 105 μM of extract. After 48 hr, the results showed that this concentration of the extract can induce apoptosis in both 4T1T and 4T1B cells. As showed in Figure 3, the level of apoptosis in the concentration of 105 μM extract was 28% in 4T1T cells and in 4T1B cells this level was 36%. This result indicated that 4T1B are more susceptible to apoptotic effects of frankincense.

### Frankincense extract significantly reduced cells migration

**Figure 4:**
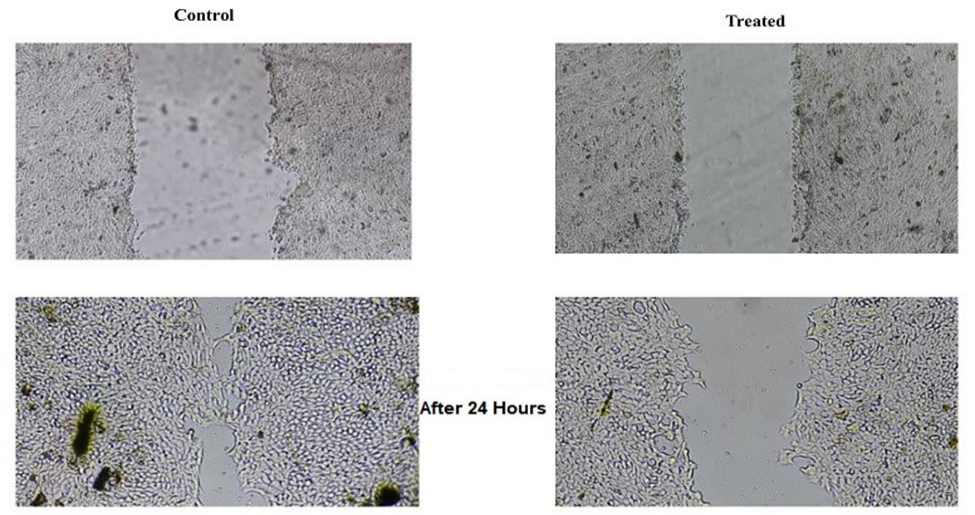
Frankincense suppressed the migration and invasion of 4T1B. The ability of migration was compared in treated and control groups based on wound healing. In frankincense treated group, the wound closure was not filled after 24 hr.

In this step, we examined cell migration in the absence or presence of the extract using the wound healing assay. In the control group, cell migration was a very dynamic process that reached 40% after 24 hr, and after 48 hr, narrow signs of the wound were observed. Our data clearly showed that treatment with the extract (105 μg/ml concentration) caused a significant inhibition of cell migration of 4T1B cells and wound closure was not complete after 24 hr (Figure 4).

### Frankincense extract inhibited enzymatic activity of MMP-9 and -2 in 4T1B

**Figure 5:**
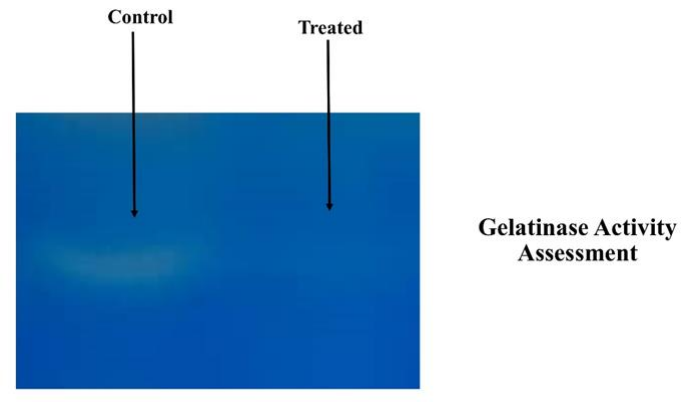
Frankincense suppressed the MMPs protein expression in 4T1B. Secretion of MMP proteins in conditioning media (CM) of 4T1B, after 48 hr treatment with the extract was not enough to be detected in zymography but MMPs secreted in CM of 4T1B in control group could create a white band on SDS-page.

Zymography was used to investigate proteolytic enzymes, especially matrix metalloproteinases (MMP)-9 and -2. The activity of MMPs was visualized as clear spots on the gel, which indicated the degradation of gelatin by these enzymes. As shown in Figure 5, secretion of MMP proteins in conditioning media (CM) of 4T1B, after 48 hr exposure with the extract, was not enough to be detected in zymography but secreted MMPs in CM of 4T1B in control group could create a white band on SDS-page.

## Discussion

In a clinical study conducted in 2007, Flavin reported that *Boswellia serrata*, as a potent inhibitor of lipoxygenase-2, can treat brain metastatic lesion of a breast cancer patient. The data collected from this research showed that *Boswellia serrata* can be a new and useful treatment option for the treatment of brain metastases in breast cancer (Flavin, 2007). To date, no study has fully addressed this issue. Evidence in the present study demonstrated that methanolic extract of frankincense gum had significant anti-tumor effect on brain metastatic cancer cells. In this study, after development of syngeneic mouse model of metastatic breast cancer, we treated the 4T1T cells (derived from primary tumor) and 4T1B cells (derived from brain metastatic lesion) with frankincense extract. Cytotoxic activities, apoptotic effects and anti-metastatic effects of the extract were determined in our study. Surprisingly, our results showed that 4T1B cells are more sensitive to anti-tumor and cytotoxic effects of frankincense extract.

The cytotoxic and apoptotic effects of frankincense (obtained from *Boswellia* species) have been confirmed in breast cancer. Suhail and colleagues measured the cytotoxic and apoptotic effects of *Boswellia sacra* essential oil on three human breast cell lines (MCF7, MDA-MB-231, and T47D). They determined that, these human breast cancer cell lines were susceptible to cytotoxic and apoptotic effects of this essential oil (Suhail et al., 2011). 

Anti-proliferative and cytotoxic effects of boswellic acids were determined in HepG2 liver cancer cell line. The results show that boswellic acids reduce cell survival and cause the death of HepG2 cells by inducing the expression of caspase-3, -8, and -9 (Liu et al., 2002). 

Yazdanpanahi et al. evaluated the cytotoxic effects of *Boswellia thurifera* gum methanolic extract on MDA-MB-231 cell line and revealed that this extract induces tumor cell death and cytotoxicity (Yazdanpanahi et al., 2014) Another research has reported that frankincense essential oil significantly reduced cell invasion and migration in MCF-7 breast cancer cell line (Isakoff, 2010). According to our studies, frankincense extract induces apoptosis and suppresses cell viability in primary and metastatic breast cancer cells.

MMPs are upregulated in breast cancer cells and lead to invasion and metastasis of cancer cells via epithelial-mesenchymal transition by breaking the physical obstacles of metastasis (Cao et al., 2019; Radisky and Radisky, 2010). As mentioned above, frankincense extract can reduce MMPs secretion in metastatic cells. Therefore, frankincense extract indirectly inhibits epithelial mesenchymal transition (EMT) and prevents cancer cells invasion.

As discussed, there are several studies about the effects of frankincense on tumor cells, but the anti-cancer effects of methanolic frankincense extract on brain metastatic breast cancer cells have not yet been studied. Several studies show that methanol and other polar extracts of plants are more efficient than non-polar extracts (Yazdanpanahi et al., 2014). According to the present study, methanolic extract of frankincense has potent anti-tumor effects on brain metastatic breast cancer cells. Thus, it can be a very effective herbal medicine for treating breast cancer and overcome its brain metastasis.
